# A New Recessive Gene Conferring Resistance Against Rice Blast

**DOI:** 10.1186/s12284-016-0120-7

**Published:** 2016-09-15

**Authors:** Zhijian Liang, Ling Wang, Qinghua Pan

**Affiliations:** State Key laboratory for Conservation and Utilization of Subtropic Agrobioresurces, Guangdong Provincial Key Laboratory for Crop Molecular Breeding, Rice Blast Research Center, South China Agricultural University, Guangzhou, 510642 People’s Republic of China

**Keywords:** *Oryza sativa*, *Magnaporthe oryzae*, *aus* rice cultivar, Recessive resistance gene

## Abstract

**Background:**

Rice blast (causative pathogen *Magnaporthe oryzae*) represents a major biotic constraint over rice production. While numerous genes for resistance have been found in both *japonica* and *indica* germplasm, as yet the diversity harbored by *aus* germplasm has not been widely exploited.

**Results:**

The blast resistance present in the *aus* type cultivar AS20-1 was shown, via an analysis of segregation in the F_2_ generation bred from a cross with the highly blast susceptible cultivar Aichi Asahi, to be due to the action of a single recessive gene, denoted *pi66*(t). The presence of *pi66*(t) gave an intermediate level control to plants infected with the blast pathogen isolate EHL0635. A bulked segregant analysis indicated that four microsatellite loci (SSRs) mapping to chromosome 3 were probably linked to *pi66*(t). Localized mapping using chromosome 3-based SSRs and Indels defined a genetic window for *pi66*(t), flanked by the markers F04-j2 and M19-i12, which physically equals to 27.7 and 49.0 kb, respectively, in the reference genomes of cultivars Nipponbare and 93–11. This physical interval does not harbor any major gene currently associated with disease resistance.

**Conclusion:**

*pi66*(t) is one of just three recessive genes controlling rice blast, and is the first major gene for resistance to be mapped to chromosome 3.

**Electronic supplementary material:**

The online version of this article (doi:10.1186/s12284-016-0120-7) contains supplementary material, which is available to authorized users.

## Background

Rice, a crop which feeds half of the world’s population, has been cultivated for at least 8,000 years (Khush 1997; The 3,000 rice genome project 2014; Travis et al. 2015). Five distinct groups of rice germplasm have long been recognized: they are referred to as *indica*, *aus*, basmati/sadri, tropical *japonica* and temperate *japonica* (The 3,000 Rice Genomes Project 2014; Travis et al. 2015). The *aus* group has developed in the north-eastern region of the Indian sub-continent, where both the climate and the growing environment are highly variable (Mahender et al. 2012; Travis et al. 2015). In recent years, *aus* germplasm has grown in importance as a source of genes for rice improvement, especially in the context of breeding for resistance/tolerance to abiotic and biotic stress (Travis et al. 2015 and references therein).

Rice blast (causative pathogen *Magnaporthe oryzae*) is a major constraint over rice production, inducing grain yield losses of up to 90 % (He et al. 2012; Singh et al. 2015). Although breeders have so far been able to rely on a number of sources of genetic resistance, the pathogen is adept at evolving new races, with the result that mongenic resistances typically break down quite rapidly (Wu et al. 2014; Singh et al. 2015; Zhang et al. 2015). To date, some one hundred rice blast resistance (*Pi*) genes have been identified, many of which have been shown to map within a cluster or even in form of a tandem array; they are dispersed on eleven of the twelve rice chromosomes (Sharma et al. 2012; Singh et al. 2015; Tanwaeer et al. 2015 and references therein). All but two of the *Pi* genes are functionally dominant (Fukuoka et al. 2009; He et al. 2012), and about 30 have been isolated: their products mostly belong to the large group of nucleotide-binding site (NBS)-leucine-rich repeat (LRR) proteins. The two exceptions are *Pid-2* and *pi21* (Chen et al. 2006b; Fukuoka et al. 2009; Liu et al. 2011). Here, a third recessive gene, denoted *pi66*(t), has been identified in the *aus* cultivar (cv.) AS20-1, and its genomic position has been defined.

## Results

### Resistance Reaction and Spectrum

Numerous differential reactions were identified among the four cvs in the five *Mo* populations, suggesting that the *Pi* gene(s) carried by the donor cv. AS20-1 could be distinguished from the other *Pi* genes with these reactions (Table 1 and Additional file 1: Table S1). Intermediate and even lower resistance frequencies were evaluated among the four cvs in the five *M. oryzae* populations, indicating that all the four *Pi* genes should be incorporated with other *Pi* genes to stand the higher level of resistance in a given cultivar, if it will be released in the five *M. oryzae* populations.

**Table 1:** Reactions shown by four rice cultivars infected by a *M. oryzae* isolate representative of each of the five Chinese populations, and the frequency of resistance exhibited among the collected isolates from each population

*Mo* population	Selected isolate	Specific reactions selected from the five *Mo* populations^a^				Resistance frequencies in the five *Mo* populations (%)^b^			
		AS20-1	Aichi Asahi	Kasalath	IRBLta2-Pi	AS20-1	Aichi Asahi	Kasalath	IRBLta2-Pi
Guangdong	CHL3417	R	R	R	S	45.0	45.0	48.3	39.7
Guangxi	EHL1622	S	S	S	R	25.0	26.7	36.7	38.3
Yunnan	EHL0210	MS	S	MS	MS	36.7	36.7	53.3	73.3
Sichuan	CHL892	MR	S	S	S	20.0	20.0	48.3	56.7
Heilongjiang	EHL1379	S	S	R	S	6.7	1.7	63.3	56.7

^a^ *R* resistant, *S* susceptible, *MS* moderately susceptible, *MR* moderately resistant

^b^Resistance frequencies were based on 60 isolates except for Kasalath and IRBLta2-Pi in the Guangdong population, in which only 58 isolates were tested

### Resistance Inheritance

**Fig. 1:**
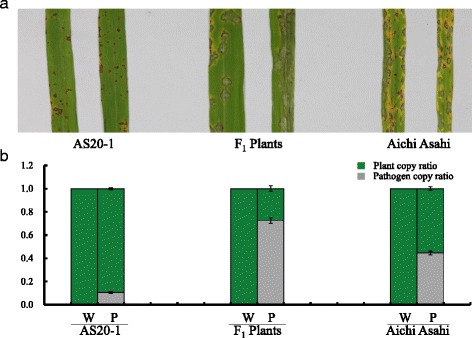
The effectiveness of *pi66*(t) to resist infection by *M. oryzae.* **a** The infection phenotype of cvs AS20-1 (resistant), Aichi Asahi (susceptible) and the cv. AS20-1 x cv. Aichi Asahi F_1_ hybrid (highly susceptible). **b** qPCR-based quantification of infection. Each bar represents the mean ± standard deviation (*n* = 3). Similar results were obtained from two biological replications each with three technical repeats. W: mock inoculation with water, P: inoculation with the pathogen isolate EHL0635

When challenged by the blast isolate EHL0635, cv. AS20-1 was scored as moderately resistance (MR), cv. Aichi Asahi as susceptible (S) and the cv. AS20-1 x cv. Aichi Asah F_1_ as highly susceptible (HS) (Fig. 1). The qPCR-based assay confirmed that the hybrid was more susceptible than cv. Aichi Asahi. The F_2_ progeny segregated as 101 R, 282 MR, 254 MS and 883 S, fitting a monogenic 1R:3S ratio when the R/MR and MS/S classes were combined (*χ*^2^ = 0.02; *P* > 0.80; Table 2). Together, these results indicated that the blast resistance expressed by cv. AS20-1 relied on homozygosity for the recessive allele of a single gene.

**Table 2:** Segregation for resistance in the F_2_ population bred from the cross cv. AS20-1 x cv. Aichi Asahi, following inoculation with the *M. oryzae* isolate EHL0635

Parents/F_2_ plants	No. of plants^a^					Segregation^b^ ration	*χ* ^2c^	*P*
	R	MR	MS	S	Total			
AS20-1	9	19	1	0	29	*na*		
Aichi Asahi	0	0	0	32	32	*na*		
F_2_ population	101	282	254	883	1520	1R:3S	0.02	>0.80

^a^ *R* resistant, *S* susceptible, *MS* moderately susceptible, *MR* moderately resistant

^b^ *na* not applicable

^c^Chi-square test using the Yates correction comparing resistance [R + MR] with susceptibility [MS + S]

### Gene Locus

**Fig. 2:**
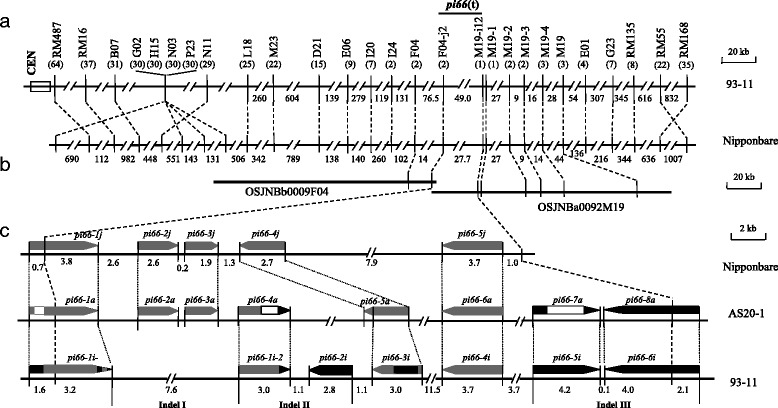
The genomic location of *pi66*(t). **a** Physical maps of the *pi66* region based on the reference sequences of cvs 93–11 and Nipponbare. The numbers shown below the map represent the physical distance in kb, those shown in parentheses represent the numbers of recombinants/gametes detected in the mapping population. **b** A BAC contig map of the *pi66*(t) region derived from the cv. Nipponbare tiling map. **c** The predicted gene content of the mapping interval harboring *pi66*(t). Three substantial Indel events, which results in six genome-specific genes presented in the region, were determined via genome comparison and P/A analyses, of which three chimeric genes presented in both 93–11 and AS20-1 genomes. Candidate genes of cvs Nipponbare, 93–11, and As20-1 were indicated with *grey*, *black*, and *blank arrows*, respectively

BSA analysis revealed that four SSR markers (RM487, RM16, RM55, and RM168) on rice chromosome 3 were candidate markers linking to the target *Pi* gene, exclusively, in the F_2_ population. The first round of linkage analysis with 750 viable F_2_ plants revealed that there were 64 and 37 recombinants, respectively, at RM487 and RM16 loci on the centromere side, 35 and 22 distinct recombinants, respectively, at RM168 and RM55 loci on the telomere side, indicating that the four candidate markers were indeed linkage markers with the target *Pi* gene (Fig. 2a). Because no major *Pi* gene had been previously identified in this region, the novel *Pi* gene in AS20-1 was designated as *pi66*(t).

Additional nine polymorphic SSR markers developed in the region defined by the flanking markers RM16 and RM55 were subjected to the second round of linkage analysis (Additional file 2: Table S2). The results showed that there were 31 to 22 recombinants detected among the seven marker loci [B07 (31), G02 (30), H15 (30), N03 (30), P23 (30), N11 (29), L18 (25), M23 (22)] on the centromere side, and only 8 distinct recombinants at RM135 locus on the telomere side (Fig. 2a). A total of 14 additional Indel markers developed in the narrower region flanked by markers M23 and RM135 were subjected to the third round of linkage analysis (Additional file 2: Table S2). The results showed that there were 15 to 2 recombinants detected among the six marker loci [D21 (15), E06 (9), I20 (7), I24 (2), F04 (2), F04-j2 (2)] on the centromere side, and 7 to 1 recombinant(s) detected among the eight marker loci [G23 (7), E01 (4), M19 (3), M19-4 (3), M19-3 (2), M19-2 (2), M19-1 (1), M19-i12 (1)] on the telomere side (Fig. 2a). The target locus, *pi66*(t), was closely flanked by F04-j2 and M19-i12, which equals to 27.7 and 49.0 kb, respectively, in the reference genomes of cvs Nipponbare and 93–11 (Fig. 2a).

### Candidate Genes

The *pi66*(t) region was represented by the two cv. Nipponbare overlapping BACs OSJNBb0009F04 and OSJNBa0092M19 (Fig. 2b). The number of genes present within this region was six in cv. Nipponbare and 14 in cv. 93–11 (Additional file 3: Table S3). Genome comparison and presence/absence (P/A) analyses revealed that there were three substantial Indel events that resulted in six genome-specific genes in the region. That is, both *pi66-2j* and *pi66-3j* in Indel I present in two genomes of cvs Nipponbare and AS20-1; *pi66-1i-2* (a duplication of *pi66-1i-1*) in Indel II, *pi66-5i* and *pi66-6i* in Indel III in that of both cvs 93–11 and AS20-1; and *pi66-2i* in Indel II in that of cv 93–11, only (Fig. 2c, Additional file 4: Figure S1). Notably, there were six transposon-like genes (*pi66-1j*, -*2j*, -*3j*, *1i-1*, *1i-2*, -*6i*), of which both *pi66-2j* and *-3j* were scattered across the entire genomes except for the target region of cv. 93–11, thereby ruling out for P/A analyis (Additional file 4: Figure S1; Additional file 3: Table S3). Furthermore, there were three chimeric genes in both 93–11 and AS20-1 genomes (Fig. 2c and Additional file 4: Figure S1). By excluding six transposon-like genes, there were three most possible candidates (*pi66-5a*, -*6a*, -*7a*) for *pi66*(t) (Fig. 2c; Additional file 3: Table S3).

## Discussion

Chinese rice breeders have to date largely ignored *aus* germplasm, even though it has acquired a growing reputation for harboring genes for resistance/tolerance to abiotic and biotic stress (Travis et al. 2015). Rather, efforts to improve *indica* have concentrated on materials developed in SE Asia, while those directed at *japonica* have relied on germplasm from Japan (Wu et al. 1991). In addition to *pi66*(t), *aus* germplasm has also yielded both *Pi16* and an allele of *Pik* (Pan et al. 1999). More recently, nine already recognized *Pi* genes have been identified as present in materials originating in NE and E India (Imam et al. 2014), which is the center of origin of *aus* germplasm. Notably, the donor of *pi66*(t) also harbors a gene conferring resistance against the brown plant hopper; this gene also lies on chromosome 3, but at some distance from *pi66*(t) (Chen et al. 2006a). Such works clearly indicated that *aus* cvs are valuable and promising genetic resources for withstanding biotic pressures including rice blast disease, and will greatly enlarge the gene pool for rice breeders.

Plant disease resistance genes have been classified into two types, the most frequent of which encode an NBS-LRR protein. Non-NBS-LRR genes encode a wide diversity of products (Chauhan et al. 2015; Olukolu et al. 2016), tend to confer partial (rather than complete) resistance and are typically more durable than the NBS-LRR type genes. The most well documented non-NBS-LRR type is barley *mlo*, a gene which encodes a G protein-coupled receptor residing in the plasma membrane (Kim et al. 2002); the gene confers durable resistance to a broad spectrum of powdery mildew races (Acevedo-Garcia et al. 2014). A second example is the wheat gene *Lr34*, which encodes an ATP-binding cassette transporter; its product protects against five distinct foliar fungal pathogens (Kratttinger et al. 2009; Chauhan et al. 2015). The maize gene *ZmWAK* encodes a plasma membrane-related receptor-like kinase; its presence has been correlated with a reduction in the incidence of head smut disease (Zuo et al. 2015). Finally, the rice gene *xa5* encodes a small subunit of the transcription factor IIA (TFIIA); this gene confers resistance against bacterial blight (Iyer-Pascuzzi 2004). Before the identification of *pi66*(t), all but two of the *Pi* genes characterized to date act as dominant alleles. The exceptions are *pi21* and *pi55.* The former gene encodes a proline-rich protein harboring a probable heavy metal-binding domain and some predicted protein-protein interaction motifs; the resistant allele differs from the wild type dominant one by two deletions affecting the latter motifs, and which are thought to be responsible for the allele's determination of non-race-specific resistance (Fukuoka et al. 2009). One of candidate genes for *pi55* encodes a protein rather similar to that encoded by *pi21*. Although a substantial number of major *Pi* genes have been intra-chromosomally mapped, *pi66*(t) is the first to be located on chromosome 3. Two quantitative trait loci mapping to this chromosome (*Os03g0122000* and *Os03g0120400*) have been associated with blast resistance (Wang et al. 2014), but both lie outside the critical RM16-RM55 interval. The *pi66* identified in the current study that is the third recessive *Pi* gene located on the virgin land, where no any known Pi protein (domain) is identifiable (Additional file 3: Table S3). It is noteworthy that the *bph19* derived from the donor cv. AS20-1 was also recognized as non-NBS-LRR resistance gene (Chen et al. 2006a). It has been argued that durable and broad-spectrum resistance may be more readily achieved by deploying non-NBS-LRR genes, perhaps in combination with NBS-LRR ones, than by attempting to stack genes which each (at least for some time) confer immunity (Fukuoka et al. 2009; Acevedo-Garcia et al. 2014; Chauhan et al. 2015; Zuo et al. 2015). This hypothesis can only be tested by exploiting genes such as *pi66*(t) in a rice breeding program.

## Conclusions

This research has confirmed that novel resistance genes against blast can be recovered from *aus* germplasm. The gene *pi66*(t) identified here is the third recessive *Pi* gene to be identified, and is also the first major *Pi* gene to be located on chromosome 3.

## Methods

### Phenotyping

The *pi66*(t) donor cv. AS20-1, along with the *Pia* carrier cv. Aichi Asahi, the *Pi36* carrier cv. Kasalath and the *Pita-2* carrier cv. IRBLta2-Pi were challenged with 60 *M. oryzae* isolates collected from each of Guangdong (GD), Guangxi (GX), Yunnan (YN), Sichuan (SC) and Heilongjiang (HLJ) provinces. Inoculation and scoring methods were adapted from those described by Pan et al. (1996, 2003). Plants were assigned a score of either 0–1 (resistant: R), 2–3 (moderately resistant: MR), 4 (moderately susceptible: MS) or 5 (susceptible: S). The frequency of resistance for each of the four cultivars within each of the five *M. oryzae* populations was calculated from [(R + MR)/(R + MR) + (MS + S)]. The typical reactions of cv. AS20-1, cv. Aichi Asahi and the cv. AS20-1 x cv. Aichi Asahi F_1_ plants were quantified using a quantitative PCR (qPCR) assay, according to the protocols previously described (Berruyer et al. 2006; Kawano et al. 2010; Zhang et al. 2015). *Oryza sativa OsUbi* (Gene ID: 4332169) and *M. oryzae Pot2* (Gene ID: 2680652) were used as the reference genes for, respectively, the host and the pathogen DNA.

### Chromosome Mapping

The donor cv. AS20-1 was crossed with the highly susceptible cv. Aichi Asahi, and their F_2_ progenies were screened for reaction to inoculation with *M. oryzae* isolate EHL0635. The F_2_ population showing monogenic segregation was regarded as the mapping population, thereby subjecting to the bulked-segregant assay (BSA) for quickly mapping chromosomal region involving the target gene. Genomic DNAs of the F_2_ plants as well as the parental plants were extracted from frozen leaves using the CTAB method. Two contrast bulks that were constructed by pooling equimolar amounts of DNAs from 10 resistant or 10 susceptible F_2_ plants. The two bulks, along with both parental DNAs, were then assayed with a set of 180 simple sequence repeat (SSR) markers (Temnykh et al. 2000, 2001), selected to span the full rice genome, following the methods given by He et al. (2012).

### Gene Mapping

Genomic map of target gene were established through three rounds of linkage analysis using genomic position-ready molecular markers (He et al. 2012). The first round was carried out with candidate markers defined by BSA for screening recombinants on both sides of the target locus. The second round was carried out with additional SSR markers in the target region flanked by the closest markers derived from the first round of linkage analysis, which were developed on the basis of reference sequence of cv. Nipponbare, except for RM135 that was adopted from the rice SSR marker maps (Temnykh et al. 2000, 2001). The third round was carried out in the recombinant progeny with insertion/deletion (Indel) markers those were developed *de novo* based on differential sequences between the two reference sequences of *japonica* cv. Nipponbare and *indica* cv. 93–11. Linkage marker search and prime designation were performed in the way essentially same way as previously described (Liu et al. 2005; Zeng et al. 2011; He et al. 2012). Genomic map of the target locus was constructed on the basis of both reference sequences.

### Candidate Gene Indentification

Candidates for *pi66*(t) were predicted based on gene annotations provided by BLASTN (www.ncbi.nlm.nih.gov/BLAST), RiceGAAS (ricegaas.dna.affrc.go.jp) and FGENSH (www.softberry.com) software. The two reference sequences proved to be rather diverse in the target region, so candidates that encode proteins with over 200 aa was validated by PCR-based presence/absence (P/A) test against the four DNAs of cvs AS20-1, Aichi Asahi, 93–11, and Nipponbare, following Zhai et al. (2011).

## Additional Files

Additional file 1: Table S1. Sampling and phenotyping information for the 300 isolates selected from five Chinese *M. oryzae* populations. (XLSX 31 kb) Additional file 2: Table S2. PCR-based markers mapping to the *pi66*(t) region. (DOCX 50 kb) Additional file 3: Table S3. The gene content of the region flanked by the *pi66-*linked markers F04-j2 and M19-i12. (DOCX 22 kb) Additional file 4: Figure S1. Presence/absence analysis of nine candidate genes. The upper panel shows the schematic gene structure used for primer design, and the lower two panels show the amplicons. 93: cv. 93–11, Ni: cv. Nipponbare, AS: cv. AS20-1, Ai: cv. Aichi Asahi, M1: size marker DL15,000; M2: size marker DL2,000. (PPTX 680 kb)
