# Supplementary material for: A New Recessive Gene Conferring Resistance Against Rice Blast
Source: Rice (N Y). 2016 Sep 15;9:47. doi: 10.1186/s12284-016-0120-7 (PMC5025421; doi:10.1186/s12284-016-0120-7)
Supplement: Additional file 2: Table S2. — PCR-based markers mapping to the pi66(t) region. (DOCX 50 kb) [file 12284_2016_120_MOESM2_ESM.docx]

Table S2 PCR-based markers mapping to the *pi66*(t) region

| **Marker^a^** | | **Type ^b^** | | **Primer sequence**  **(5' → 3') ^c^** | **Source^d^** | | **Annealing**  **temp (℃) ^e^** | **Expected**  **size (bp) ^f^** |
| --- | --- | --- | --- | --- | --- | --- | --- | --- |
| **First round of linkage analysis (BSA)** | | | | | | | | |
| RM487 | | SSR | | F: TTTCTCGAACGCAGGAGAAC | | OSJNBb0056O10 | 66 | 175 |
|  | |  | | R: GCTAGGAACATCAACCCGAG | |  |  |  |
| RM16 | | SSR | | F: CGCTAGGGCAGCATCTAAA | | OSJNBb0007E22 | 60 | 167 |
|  | |  | | R: AACACAGCAGGTACGCGC | |  |  |  |
| RM55 | | SSR | | F: CCGTCGCCGTAGTAGAGAAG | | [OSJNBa0078A17](http://www.ncbi.nlm.nih.gov/clone/?term=OSJNBa0078A17) | 60 | 145 |
|  | |  | | R: TCCCGGTTATTTTAAGGCG | |  |  |  |
| RM168 | | SSR | | F: TGCTGCTTGCCTGCTTCCTTT | | [OSJNBb0017F17](http://www.ncbi.nlm.nih.gov/clone/?term=OSJNBb0017F17) | 65 | 93 |
|  | |  | | R: GAAACGAATCAATCCACGGC | |  |  |  |
| **Second round of linkage analysis (elementary mapping)** | | | | | | | | |
| P23 | | SSR | | F: GACTGATAGCTGCAATGTG | | OSJNBb0016P23 | 58 | 195 |
|  | |  | | R: TGTAGGAAGTTTCATGGACT | |  |  |  |
| N11 | | SSR | | F: TCTTGGCATGTATGGATGG | | [OSJNBb0042N11](http://www.ncbi.nlm.nih.gov/clone/?term=OSJNBb0042N11) | 58 | 160 |
|  | |  | | R: GAAGGAGAAAGGGAGAATGA | |  |  |  |
| B07 | | SSR | | F: GCTGTCATCACTACTACCC | | [OSJNBb0111B07](http://www.ncbi.nlm.nih.gov/clone/?term=OSJNBb0111B07) | 54 | 185 |
|  | |  | | R: ATCGCATGTAGAGTCTTAGG | |  |  |  |
| G02 | | SSR | | F: CACCACGCTACACCAGTA | | [OSJNBb0070G02](http://www.ncbi.nlm.nih.gov/clone/?term=OSJNBb0070G02) | 55 | 117 |
|  | |  | | R: GAAGGCTGTGAGACTTTCG | |  |  |  |
| N03 | | SSR | | F: ACCACATCCACCGCTAAT | | [OSJNBa0010N03](http://www.ncbi.nlm.nih.gov/clone/?term=OSJNBa0010N03) | 55 | 92 |
|  | |  | | R: GAACCTTCCTCCTTTATACCTT | |  |  |  |
| H15 | | SSR | | F: CTCCTCTGATGCTTCCATT | | [OSJNBa0066H15](http://www.ncbi.nlm.nih.gov/clone/?term=OSJNBa0066H15) | 57 | 181 |
|  | |  | | R: TCACACTGCTCTTCAACTG | |  |  |  |
| L18 | | SSR | | F: GCATTGCGATCTGCGAAA | | [OSJNBa0007L18](http://www.ncbi.nlm.nih.gov/clone/?term=OSJNBa0007L18) | 54 | 98 |
|  | |  | | R: ACACGTATGCGAGATAAGGA | |  |  |  |
| M23 | | SSR | | F: CTCATTCCATCCTGTGTAATAC | | [OSJNBa0093M23](http://www.ncbi.nlm.nih.gov/clone/?term=OSJNBa0093M23) | 56 | 233 |
|  | |  | | R: GTCCTAGATATGCTCATCCAA | |  |  |  |
| RM135 | | SSR | | F: CTCTGTCTCCTCCCCCGCGTCG | | [OSJNBb0024N19](http://www.ncbi.nlm.nih.gov/clone/?term=OSJNBb0024N19) | 60 | 130 |
|  | |  | | R: TCAGCTTCTGGCCGGCCTCCTC | |  |  |  |
| **Third round of linkage analysis (fine mapping)** | | | | | | | | |
| D21 | | Indel | | F: GAAGCCTAGCATACCACAA | | [OSJNBa0034D21](http://www.ncbi.nlm.nih.gov/clone/?term=OSJNBa0034D21) | 57 | 158 |
|  | |  | | R: CATAACCTCGTCGTCCTC | |  |  |  |
| E06 | | Indel | | F: CTCCTGCCGTCATGTTAT | | OSJNBa0056E06 | 60 | 410 |
|  | |  | | R: CAACTCGTGTCCAACTGT | |  |  |  |
| I20 | | Indel | | F: GCACACCAACTCACTCTT | | [OSJNBb0113I20](http://www.ncbi.nlm.nih.gov/clone/?term=OSJNBb0036M02) | 60 | 376 |
|  | |  | | R: CCGTTTCGTCTATGTTCATT | |  |  |  |
| I24 | | Indel | | F: TATGCTTGTACTCCTAAAGGAACC | | [OSJNBa0035I24](http://www.ncbi.nlm.nih.gov/clone/?term=OSJNBa0035I24) | 62 | 160 |
|  | |  | | R: CATCATATCTGGTGTGGCTTG | |  |  |  |
| F04 | | Indel | | F: GTTCTGTGAAGAGTACAAGTAG | | [OSJNBb0009F04](http://www.ncbi.nlm.nih.gov/clone/?term=OSJNBb0009F04) | 58 | 119 |
|  | |  | | R: AGAAAGCATCACCTCAAACT | |  |  |  |
| F04-j2 | | Indel | | F: GGGTAACGGGCATATTTGA | | [OSJNBb0009F04](http://www.ncbi.nlm.nih.gov/clone/?term=OSJNBb0009F04) | 60 | 255 |
|  | |  | | R: AGCTAGAGGAGGAAGAAGACATAT | |  |  |  |
| M19-i12 | | Indel | | F: TGGATCGAATGTCTGGAT | | [OSJNBa0092M19](http://www.ncbi.nlm.nih.gov/clone/?term=OSJNBa0092M19) | 58 | 132 |
|  | |  | | R: GGGAGTGTGACGAGTTT | |  |  |  |
|  | |  | |  | |  |  |  |
| **Table S2** Continued | | | |  | |  |  |  |
| **Marke^a^** | **Type ^b^** | | **Primer sequence**  **(5' → 3') ^c^** | | | **Source^d^** | **Annealing**  **temp (℃) ^e^** | **Expected**  **size (bp) ^f^** |
| M19-1 | Indel | | F: CATCTGAGTTAATCGTAGGTAG | | | [OSJNBa0092M19](http://www.ncbi.nlm.nih.gov/clone/?term=OSJNBa0092M19) | 62 | 178 |
|  |  | | R: TAAGGTGGTGCCATCTGA | | |  |  |  |
| M19-2 | Indel | | F: AACATACGCTATTGCGGCTC | | | [OSJNBa0092M19](http://www.ncbi.nlm.nih.gov/clone/?term=OSJNBa0092M19) | 60 | 101 |
|  |  | | R: AAGATGGTTCGCGCTACC | | |  |  |  |
| M19-3 | Indel | | F: AGGGAATACCGTACACGC | | | [OSJNBa0092M19](http://www.ncbi.nlm.nih.gov/clone/?term=OSJNBa0092M19) | 60 | 111 |
|  |  | | R: CGACTGATGGGCTGATTAC | | |  |  |  |
| M19-4 | Indel | | F: GGCAAGACAAGTTTGGTAT | | | [OSJNBa0092M19](http://www.ncbi.nlm.nih.gov/clone/?term=OSJNBa0092M19) | 60 | 113 |
|  |  | | R: GAATCTCCATCCATAATTGG | | |  |  |  |
| M19 | Indel | | F: AGTTACCTCCATCCTGTTG | | | [OSJNBa0092M19](http://www.ncbi.nlm.nih.gov/clone/?term=OSJNBa0092M19) | 60 | 172 |
|  |  | | R: AGCCGTAGTCAATTCTCTAA | | |  |  |  |
| E01 | Indel | | F: GAACCTATAAACTTGTGGCA | | | [OSJNBa0067E01](http://www.ncbi.nlm.nih.gov/clone/?term=OSJNBa0067E01) | 60 | 169 |
|  |  | | R: GAAGGCATTTCCAACAGAT | | |  |  |  |
| G23 | Indel | | F: AAGATTATGCGACGGACAA | | | [OSJNBa0003G23](http://www.ncbi.nlm.nih.gov/clone/?term=OSJNBa0003G23) | 52 | 115 |
|  |  | | R: GGCGGGTTCTATTTACTTTC | | |  |  |  |
| **Presence and absence analysis of candidate genes ^g^** | | | | | | | | |
| 1j-1 |  | | F: TCTTACTGCTCCATCCTTCAAACACTTC | | | [OSJNBa0092M19](http://www.ncbi.nlm.nih.gov/clone/?term=OSJNBa0092M19) | 68 | 4205 |
|  |  | | R:CGTACTTTCTCCTCACTATTGTTCCCTCC | | |  |  |  |
| 1j-2 |  | | F: TCGGTGATGATTATGCTTCCCTATTA | | | [OSJNBa0092M19](http://www.ncbi.nlm.nih.gov/clone/?term=OSJNBa0092M19) | 68 | 3595 |
|  |  | | R: TCATCAATCATTACGCCTCCACCAGTCA | | |  |  |  |
| 4j-1 |  | | F:AGGTGACTGGTAGGAATAGGAGGCATG | | | [OSJNBa0092M19](http://www.ncbi.nlm.nih.gov/clone/?term=OSJNBa0092M19)  [OSJNBa0092M19](http://www.ncbi.nlm.nih.gov/clone/?term=OSJNBa0092M19) | 68 | 3030 |
|  |  | | R:GCTGTGGGAGGAGAATTGGACGTGGAC | | |  |  |  |
| 4j-2 |  | | R:GGTGCAGAAATGATTTAATTGTACGGCG | | |  | 67 | 2245 |
| 5j-1 |  | | F: ACGAGGCATTGTCATATTCATAGCA | | | [OSJNBa0092M19](http://www.ncbi.nlm.nih.gov/clone/?term=OSJNBa0092M19) | 66 | 3592 |
|  |  | | R: GCAAGTGTTCTCATCAAGGACTAGG | | |  |  |  |
| 5j-2 |  | | F: TGCCAGTAGACTCCATTGTAACGAT | | | [OSJNBa0092M19](http://www.ncbi.nlm.nih.gov/clone/?term=OSJNBa0092M19) | 66 | 4334 |
|  |  | | R: CCTCCTACACAGTCACATAGATAATTG | | |  |  |  |
| 1i1-1 |  | | F: TCCGTGTCCAATGTTTGACCGTCCGT | | | I genome | 68 | 4971 |
|  |  | | R: CTAGATTAGAGTGAAAATTCGCCGACA | | |  |  |  |
| 1i2-1 |  | | F:ACATGGTTGTTATGATTAAGGTGGTGGT | | | I genome  I genome | 63 | 3061 |
|  |  | | R: GCAGGAAGGGAACAATGGGTTAGTAAT | | |  |  |  |
| 1i2-2 |  | | R:GAGAGGTGGATAGGCGAGTTTATATAGG | | |  | 68 | 2516 |
| 2i-1 |  | | F: AGGCAGTAGAAGGCATAGGCAGTAGTA | | | I genome | 69 | 2811 |
|  |  | | R:TGTCGGTGCGTAATAACATAAGGTAGGA | | |  |  |  |
| 2i-2 |  | | R: ACGCATCTGACAGACACGCCTAATC | | | I genome | 68 | 2309 |
| 3i-1 |  | | F:CCTCTGGCTGCACCCGTTAATCTACACT | | | I genome  I genome | 63 | 3609 |
|  |  | | R: CACCAGGTACGTATATACTACTCCATC | | |  |  |  |
| 3i-2 |  | | R: GAGGCAAGCTGATGGATTTCTATTCGTT | | |  | 63 | 2667 |
| 5i-1 |  | | F: TTGGGAATGGTTTCAGTGCTGGTTGGAT | | | I genome | 68 | 4157 |
|  |  | | R: GGTTATGGATGACCCGATTGTGGGCCTT | | |  |  |  |
| 5i-2 |  | | F:GGCAAGGAAGTCGCTGGCTATTGAATG | | | I genome | 69 | 2353 |
|  |  | | R:TGGTCGGTCTGGTCGAAGGTGAGGGAA | | |  |  |  |
|  |  | |  | | |  |  |  |

| **Table S2** Continued | | | | | | |
| --- | --- | --- | --- | --- | --- | --- |
| **Marke^a^** | **Type ^b^** | **Primer sequence**  **(5' → 3') ^c^** | **Source^d^** | **Annealing**  **temp (℃) ^e^** | | **Expected**  **size (bp) ^f^** |
| 6i-1 |  | F:ACCCTCGAAGCTGTGGACGATGTGG | I genome | | 68 | 4972 |
|  |  | R:GGAGGCTGGGCATGGGCACGGGGAAG |  |  |  |  |
| 6i-2 |  | F: TGACGTGCCACCCCTCCCTCTGTCTCCCC | I genome | | 62 | 3356 |
|  |  | R:TCCCCTTACCTTCCTTCCTCCATCCCTGTT |  |  |  |  |

^a^ Molecular markers were summarized based on three rounds of linkage analysis, as well as presence and absence analysis of candidate genes. Markers with prefix RM were taken from the GRAMENE website (http://www.gramene.org), and the others were developed in this study those were prefixed with the last triple codes of artificial chromosomes of the reference cv. Nipponbare.

^b^ SSR, simple sequence repeat; Indel, insertion/deletion.

^c^ F, forward; R, reverse.

^d^ OSJNB, BAC clones of the reference cv. Nipponbare; I genome, Oryza_indica.ASM465v1.31.chromosome.3 of the reference cv. 93-11.

^e^ All PCR runs began with one cycle at 94°C for 3 min, followed by 35 cycles at 94 °C for 30 s, 52~62 °C for 30 s, and 72 °C for 1 min; with a final extension at 72 °C for 5 min.

^f^ Applicants were separated by electrophoresis on 10 % polyacrylamide gels or 1% agarose gels.

^g^ Genomic position-specific candidate genes that encode products of at least 200 residues, which were predicted based on both the reference sequences of cvs Nipponbare and 93-11, were selected for presence and absence analysis (also see Table S2).
