# Supplementary material for: A New Recessive Gene Conferring Resistance Against Rice Blast
Source: Rice (N Y). 2016 Sep 15;9:47. doi: 10.1186/s12284-016-0120-7 (PMC5025421; doi:10.1186/s12284-016-0120-7)
Supplement: Additional file 3: Table S3. — The gene content of the region flanked by the pi66-linked markers F04-j2 and M19-i12. (DOCX 22 kb) [file 12284_2016_120_MOESM3_ESM.docx]

Table S3. The gene content of the region flanked by the *pi66*(t)*-*linked markers F04-j2 and M19-i12

| **Code ^a^** | **Annotated gene ^b^** | **Predicted protein function ^c^** | **Amino acid** |
| --- | --- | --- | --- |
| The reference sequence of cv. Nipponbare | | |  |
| *pi66-1j* | Os03g47310 | Ribosome inactivating protein with transposase-associates domain | 951 |
| *pi66-2j* | Os03g47320 | Putative transposon protein | 693 |
| *pi66-3j* | Os03g47330 | Putative transposon protein | 307 |
| *pi66-4j* |  | Hypothetical protein | 273 |
| J1 | Os03g47350 | Expressed protein | 131 |
| *pi66-5j* | Os03g47360 | Expressed protein | 342 |
| The reference sequence of cv. 93-11 | | |  |
| *pi66-1i-1* |  | Ribosome inactivating protein with transposase-associates domain | 929 |
| I1 |  | VQ domain containing protein | 143 |
| I2 |  | Hypothetical protein | 144 |
| I3 |  | Hypothetical protein | 179 |
| *pi66-1i-2* |  | Ribosome inactivating protein with transposase-associates domain | 374 |
| *pi66-2i* |  | Hypothetical protein | 396 |
| *pi66-3i* |  | Hypothetical protein | 273 |
| I4 |  | Hypothetical protein | 89 |
| I5 |  | Hypothetical protein | 137 |
| I6 |  | Hypothetical protein | 132 |
| I7 |  | Hypothetical protein | 189 |
| *Pi66-4i* |  | Hypothetical protein | 397 |
| *Pi66-5i* |  | Hypothetical protein | 916 |
| *pi66-6i* |  | Putative gypsy type transposon, Hypothetical protein | 1054 |

^a^ A gene encoding over 200 aa was recognized as the posible candidate gene for *pi66*(t), thereby assigning as *pi66-1j*, *pi66-1i* and so on. *J*, *japonica* one derives from cv. Niponbare; *I*, *indica* one from cv. 93-11.

^b^ Annotated genes were adopted from the Rice Genome Annotation Project (http://rice.plantbiology.msu.edu).

^c^ Functions of the candidate genes were predicted by the RiceGAAS (http://ricegass.dna.affrc.go.jp), GENSCAN (http://genes.mit.edu/GENSCAN.html), and FGENSH (http://linux1.softberry.com/berry.phtml) software.
