# Supplementary material for: A New Recessive Gene Conferring Resistance Against Rice Blast
Source: Rice (N Y). 2016 Sep 15;9:47. doi: 10.1186/s12284-016-0120-7 (PMC5025421; doi:10.1186/s12284-016-0120-7)
Supplement: Additional file 4: Figure S1. — Presence/absence analysis of nine candidate genes. The upper panel shows the schematic gene structure used for primer design, and the lower two panels show the amplicons. 93: cv. 93–11, Ni: cv. Nipponbare, AS: cv. AS20-1, Ai: cv. Aichi Asahi, M1: size marker DL15,000; M2: size marker DL2,000. (PPTX 680 kb) [file 12284_2016_120_MOESM4_ESM.pptx]

## Slide 1
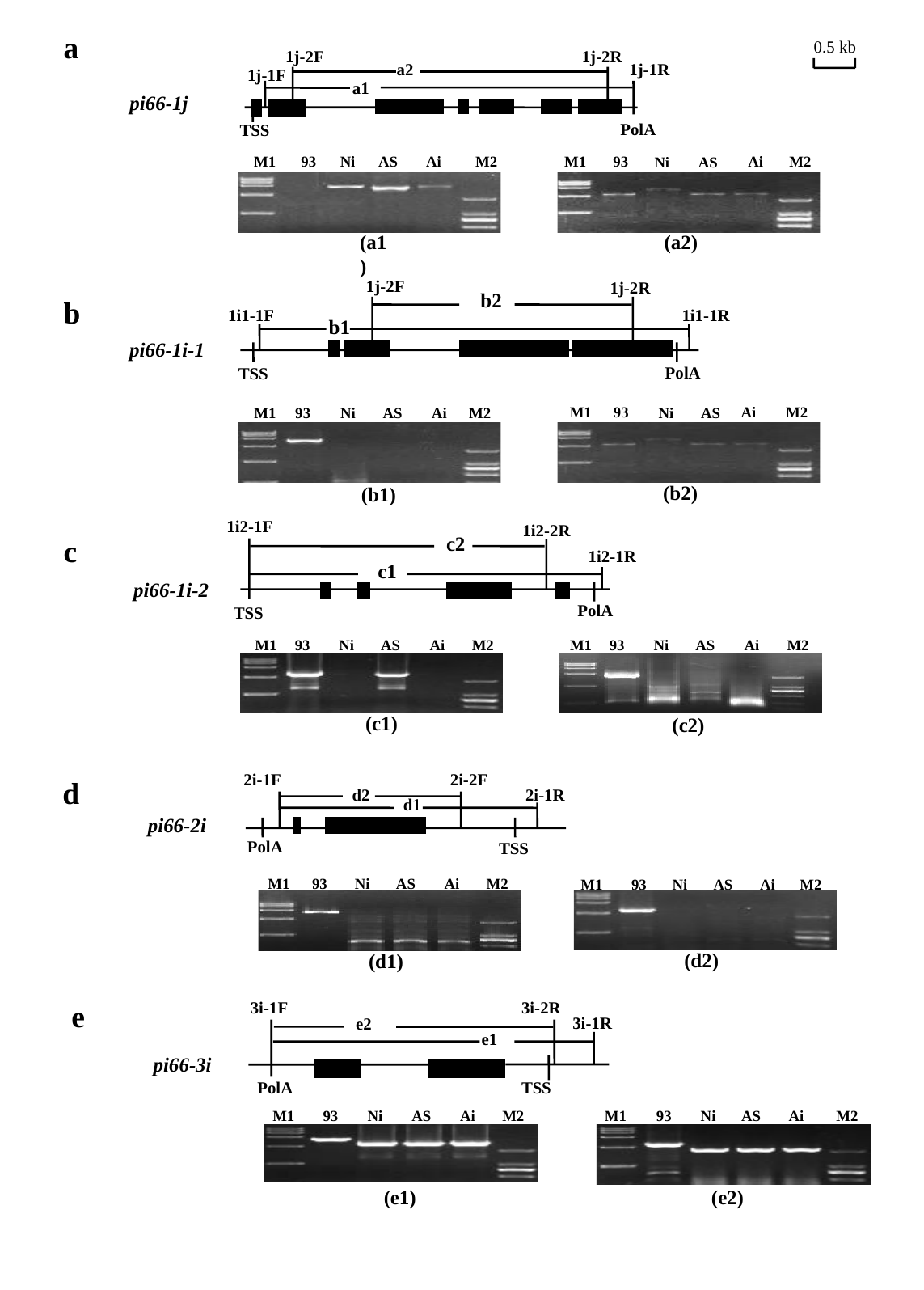

a
1j-2R
1j-2F
a2
1j-1R
1j-1F
a1
PolA
TSS
pi66-1j
M1
93
Ni
AS
Ai
M2
M1
93
Ai
M2
Ni
AS
(a1)
(a2)
0.5 kb
1j-2F
1j-2R
b2
1i1-1F
1i1-1R
b1
PolA
TSS
b
pi66-1i-1
M1
93
Ai
M2
Ni
AS
M1
93
Ai
M2
Ni
AS
(b2)
(b1)
1i2-1F
1i2-2R
c2
1i2-1R
c1
PolA
TSS
c
pi66-1i-2
M1
93
Ai
M2
Ni
AS
(c1)
M1
93
Ai
M2
Ni
AS
(c2)
2i-1F
2i-2F
2i-1R
d2
d1
PolA
TSS
d
pi66-2i
M1
93
Ai
M2
Ni
AS
(d1)
M1
93
Ai
M2
Ni
AS
(d2)
3i-1F
3i-2R
e
3i-1R
e2
e1
pi66-3i
n
PolA
TSS
M1
93
Ai
M2
Ni
AS
(e1)
M1
93
Ai
M2
Ni
AS
(e2)

## Slide 2
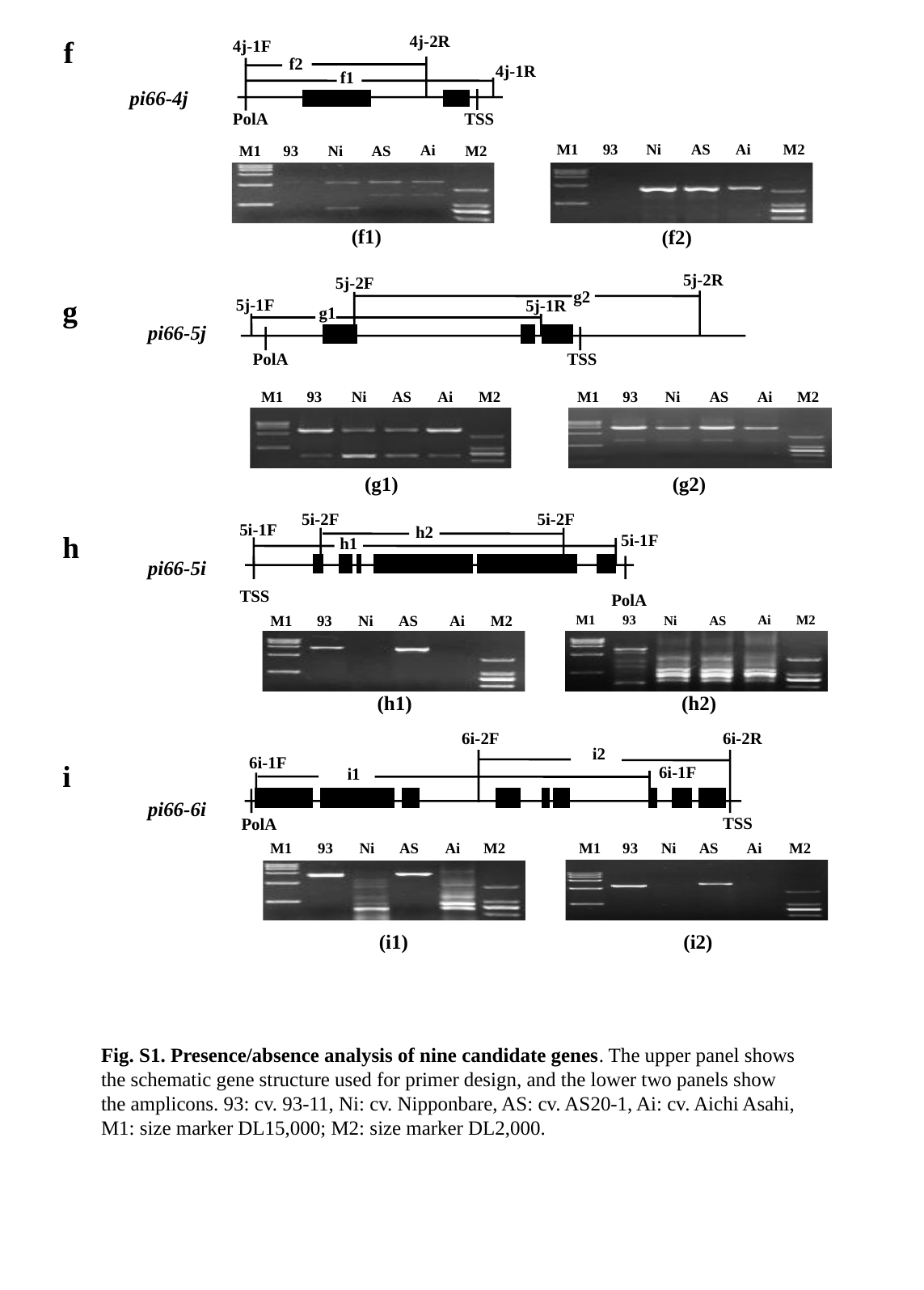

4j-2R
4j-1F
f2
4j-1R
f1
PolA
TSS
f
pi66-4j
M1
93
Ni
AS
Ai
M2
Ai
M1
93
Ni
AS
M2
(f1)
(f2)
5j-2R
5j-2F
g2
5j-1F
5j-1R
g1
PolA
TSS
g
pi66-5j
M1
93
Ai
M2
M1
93
Ai
M2
Ni
AS
Ni
AS
(g1)
(g2)
5i-2F
5i-2F
5i-1F
h2
5i-1F
h1
TSS
PolA
h
pi66-5i
M1
93
Ai
M2
Ni
AS
M1
93
Ai
M2
Ni
AS
(h1)
(h2)
6i-2F
6i-2R
i2
6i-1F
6i-1F
i1
TSS
PolA
i
pi66-6i
M1
93
Ai
M2
Ni
AS
M1
93
Ai
M2
Ni
AS
(i1)
(i2)
Fig. S1. Presence/absence analysis of nine candidate genes. The upper panel shows the schematic gene structure used for primer design, and the lower two panels show the amplicons. 93: cv. 93-11, Ni: cv. Nipponbare, AS: cv. AS20-1, Ai: cv. Aichi Asahi, M1: size marker DL15,000; M2: size marker DL2,000.
